# Response to the “letter to the editor” by Sani Rachman Soleman et al., “spatiotemporal association of low birth weight with Cs-137 deposition at the prefecture level in Japan after the Fukushima nuclear power plant accidents”

**DOI:** 10.1186/s12940-020-00661-3

**Published:** 2020-11-25

**Authors:** Hagen Scherb, Keiji Hayashi

**Affiliations:** 1 Helmholtz Zentrum München, German Research Center for Environmental Health, Institute of Computational Biology, Ingolstädter Landstr. 1, D-85764 Neuherberg, Germany; 2 Hayashi Children’s Clinic, 4-6-11-1F Nagata, Joto-ku Osaka-Shi, Osaka, 536-0022 Japan

**Keywords:** Radiation-induced genetic effects, Ecological confounding, Temporal pattern of effects, Restricted and weighted ordinary regression

## Abstract

We thank Sani Rachman Soleman et al. for three specific points of criticism concerning our investigation of the ecological association between low birth weight (LBW) and radioactive contamination in Japan after the Fukushima Daiichi Nuclear Power Plant (FDNPP) accidents:

1. *Ecological variables are not justified enough to adjust potential confounding.*
2. *The spatiotemporal regression model does not consider temporal reduction in radiation dose rate.*
3. *Dose-response plot between dose rates and odds ratios overestimates R*^*2*^ *and underestimates p-value.*

This criticism is a good starting point to explain some of the technical backgrounds of our approach in more detail.

Dear Editors,

We thank Sani Rachman Soleman et al. [1] for three specific points of criticism concerning our investigation of the ecological association between low birth weight (LBW) and radioactive contamination in Japan after the Fukushima Daiichi Nuclear Power Plant (FDNPP) accidents [2]:

1. *Ecological variables are not justified enough to adjust potential confounding.*
2. *The spatiotemporal regression model does not consider temporal reduction in radiation dose rate.*
3. *Dose-response plot between dose rates and odds ratios overestimates R*^*2*^ *and underestimates p-value.*

This criticism is a good starting point to explain some of the technical backgrounds of our approach in more detail.

## Ecological variables and confounding

Soleman et al. criticize our method for ‘*not enough control of individual variations of the LBW*’. Many epidemiological investigations of etiology are observational. In ecological studies, the unit of observation is not the individual but the population, e.g., the populations of the 47 Japanese prefectures. It is therefore not possible to adjust our regression models with individual-level variables, say the individual smoking behavior, e.g., the tobacco consumption of all pregnant women in Japan from 1995 to 2018. Such data does not exist. However, should the LBW risk factor ‘smoking behavior’ vary between and within the prefectures and by time then our spatiotemporal method automatically adjusts for this ecological confounding by smoking, since our method allows for prefecture-specific temporal base-line trends of the annual LBW proportions. We described our spatiotemporal logistic regression methodology in detail in [3]. What we of course cannot exclude is the (theoretical) possibility that the smoking behavior and the birth weight are associated with the Cs-137 deposition in the prefectures after Fukushima. However, this is a less parsimonious hypothesis as it would require a certain differential link between prefectures and consumption of cigarettes by pregnant women only from 2012 onward but not before 2012.

Our logistic regression analyses included several factors possibly impacting birth weight at the population level, for example population density and physician density, which might influence women’s health behavior via frequency or intensity of private and/or professional pregnancy counseling. After 2011, the effects of earthquake, tsunami, and nuclear accidents were added to those general factors. There are two main types: the direct effects of earthquake and tsunami and the long-term effects of the radioactive contamination. Fig. 6 in [2] compares LBW in the three radiologically contaminated and heavily earthquake and tsunami impacted prefectures Fukushima, Iwate, and Miyagi with LBW in the similarly contaminated but lesser immediately affected prefectures Ibaraki, Tochigi, and Yamagata. Because of the similarity of effects in both regions after 2011, the impacts of earthquake and tsunami are expected to negligibly contribute to the abrupt long-term LBW increases in those 6 highest affected prefectures from 2012 onward. A more direct proof that radiation damage is most likely the cause of the increase in LBW from 2012 onward is that the levels of the increases in LBW starting in 2012 correlate linearly with the intensity of radiation exposure across the prefectures of Japan, see Fig. 5 in [2]. This finding was confirmed by Soleman et al. in their Fig. 1 and in their according schematic analyses [1].

**Fig. 1:**
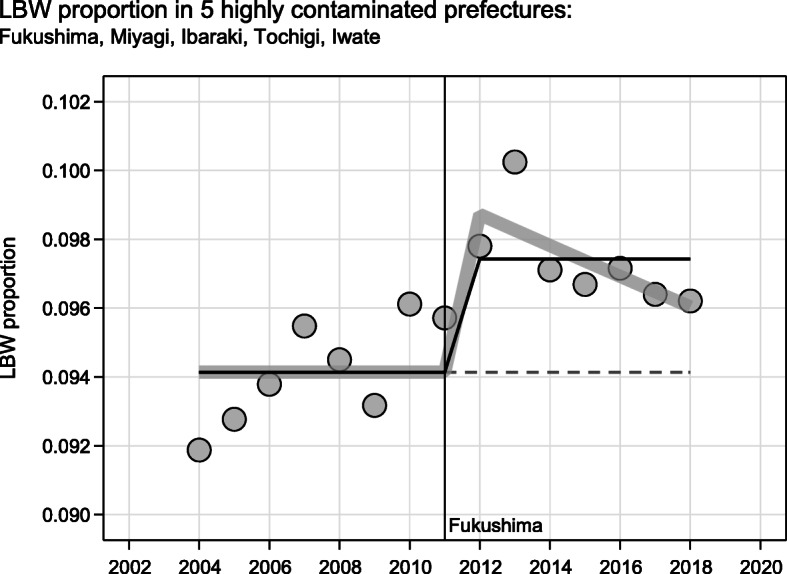
Low birth weight (LBW) proportion in the 5 highly contaminated prefectures Fukushima, Miyagi, Ibaraki, Tochigi, Iwate 2004 to 2018; two variants of logistic regression models allowing for a change-point from 2012 onward; (1) black line: simple jump in 2012 OR 1.039, (1.021, 1.057), *p*-value < 0.0001; (2) gray line: jump 2012 OR 1.059, (1.029, 1.091), *p*-value < 0.0001, interaction of jump with time OR 0.995, (0.989, 1.001), p-value 0.1075

## Neglect of the temporal reduction of the dose-rate

As Soleman SR et al. pointed out, the radiation dose is decreasing, but at the same time, it is certain that it remains in the long-term. Therefore, many Fukushima people are still forced to evacuate. Furthermore, radiation detriment is characterized by the fact that damage continues to occur for a long time after exposure. It is clear from the Life Span Study in Hiroshima and Nagasaki that not only carcinogenesis but also heart disease, respiratory disorders, and digestive disorders continue to occur for a long period of time [4]. Similarly, numerous studies report reproductive detriment concerning, e.g., stillbirths, perinatal deaths, birth defects, and chromosome aberrations demonstrating that radiation injury persisted for many years [5–17]. The elevation of LBW over long periods, despite declining radiation doses, is a hallmark of radiation injury.

In their second criticism, Soleman et al. overlooked that our estimated increase of the LBW proportion after 2011 is only the average of an effect without respect to and quantification of any temporal pattern of the LBW increase after Fukushima. To illustrate this, we analyzed the scenario of our Fig. 4D [2] concerning Fukushima, Miyagi, Ibaraki, Tochigi, and Iwate over a symmetrical period (2004 to 2018) allowing

- for a simple jump from 2012 onward, and
- for the interaction of this jump with time (technically speaking).

For according point estimates, interval estimates, and *p*-values of these analyses see Fig. 1 of this letter. It shows that the increase in LBWp decreases with time, but this decrease is not significant (p-value 0.1075) due to insufficient statistical power provided by the scenario of Fig. 4D [2]. Therefore, this decrease of the increase in the LBWp in the five highly contaminated prefectures after Fukushima is also compatible with a constant effect, at least over the 7-year period 2012 to 2018.

## Overestimating of R^2^ and underestimating *p*-value

Soleman et al. state: ‘*We found an overestimation of R*^*2*^ *and underestimation of p-value of the regression in Figure 5 of the article*’. However, in their re-analyses they overlooked that we applied variance weighted regression, which they did not. Soleman et al. could have easily performed a variance weighted regression since the confidence limits contained in our Table 2 [2] are equivalent to providing the corresponding standard errors of the 2012 jump ORs. As explicitly emphasized in [2], our Fig. 5 only served to ‘*generalize and visualize the effects seen in Figure 4*’ [2]. Accusing us of ‘*manipulation’* in combining the 37 lesser exposed prefectures to reduce the scatteredness in Fig. 5, Soleman et al. again overlook that the estimate, standard errors, and *p*-values of the variance weighted regressions (of the 37 combined and the 10 prefectures vs. the 47 individual prefectures) are practically the same up to minor deviations, see Table 1. Though not essential, restricted regression in these data is justified as the Cs-137 contamination of the prefectures is nil or negligible before the nuclear accidents. Note also that a high coefficient of determination R^2^ does not mean that it is a “good” model and a low coefficient of determination does not mean that it is a “bad” model. This is a well known fact, e.g., demonstrated by Anscombe in 1973 [18].

**Table 1:** Pertinent metrics according to variance weighted linear regression of the prefecture-specific 2012 jump ORs in LBWp trends on μSv/h for the data of Table 2 and Table 3 in [2] by regression type and prefecture stratification vs. no stratification

Regression type	variance weighted linear regression of 2012 jump OR in LBWp on μSv/h	37 not or lesser contaminated prefectures combined (***n*** = 11)	individual prefectures (***n*** = 47)
**un-restricted regression**	estimate	0.0847	0.0831
	standard error	0.0248	0.0313
	p-value	0.0078	0.0110
	R^2^	0.5637	0.1353
**restricted regression** **OR = 1**	estimate	0.1026	0.1060
	standard error	0.0156	0.0235
	p-value	< 0.0001	< 0.0001
	R^2^	0.9998	0.9989
	restriction p-value	0.3762	0.2744

In summary, we reject all criticisms by Soleman et al. [1] for the following reasons:

- Ecological variables may well serve to adjust ecological models.
- The estimation of an overall radiation effect does not necessarily require the specific temporal pattern of the effect.
- Questioning our variance weighted linear regression based on their un-weighted regression is comparing apples and pears and indicates misunderstanding of important messages of our article in several respects.

Sincerely,

Hagen Scherb and Keiji Hayashi.

## Data Availability

The employed data has exclusively been published previously and/or it is contained in the Tables and in the Figures included in this paper.

## Abbreviations

95%-CI or (.,.): 95%-confidence interval
FDNPP: Fukushima Daiichi Nuclear Power Plant
LBW: Low birth weight
LBWp: LBW prevalence or proportion
OR: Odds Ratio
SAS: Statistical Analysis System, software produced by SAS Institute Inc.
